# Timely delivery of laboratory efficiency information, Part I: Developing an interactive turnaround time dashboard at a high-volume laboratory

**DOI:** 10.4102/ajlm.v9i2.947

**Published:** 2020-04-29

**Authors:** Naseem Cassim, Manfred E. Tepper, Lindi M. Coetzee, Deborah K. Glencross

**Affiliations:** 1 National Health Laboratory Service (NHLS), Johannesburg, South Africa; 2 Department of Molecular Medicine and Haematology, Faculty of Health Sciences, University of the Witwatersrand, Johannesburg, South Africa

**Keywords:** Turn-around time, laboratory efficiency, interactive dashboard, indicators, performance assessment

## Abstract

**Background:**

Mean turn-around time (TAT) reporting for testing laboratories in a national network is typically static and not immediately available for meaningful corrective action and does not allow for test-by-test or site-by-site interrogation of individual laboratory performance.

**Objective:**

The aim of this study was to develop an easy-to-use, visual dashboard to report interactive graphical TAT data to provide a weekly snapshot of TAT efficiency.

**Methods:**

An interactive dashboard was developed by staff from the National Priority Programme and Central Data Warehouse of the National Health Laboratory Service, Johannesburg, South Africa, during 2018. Steps required to develop the dashboard were summarised in a flowchart. To illustrate the dashboard, one week of data from a busy laboratory for a specific set of tests was analysed using annual performance plan TAT cut-offs. Data were extracted and prepared to deliver an aggregate extract, with statistical measures provided, including test volumes, global percentage of tests that were within TAT cut-offs and percentile statistics.

**Results:**

Nine steps were used to develop the dashboard iteratively with continuous feedback for each step. The data warehouse environment conformed and stored laboratory information system data in two formats: (1) fact and (2) dimension. Queries were developed to generate an aggregate TAT data extract to create the dashboard. The dashboard successfully delivered weekly TAT reports.

**Conclusion:**

Implementation of a TAT dashboard can successfully enable the delivery of near real-time information and provide a weekly snapshot of efficiency in the form of TAT performance to identify and quantitate bottlenecks in service delivery.

## Introduction

Turn-around time (TAT) is an important performance indicator for a laboratory service. It refers to the time from first registration in a laboratory to a result released on the laboratory information system (LIS).^1^ Historically, within the National Health Laboratory Service (NHLS) of South Africa, TAT reporting was provided in annual and quarterly static management reports generated by the LIS, TrakCare,^2^ which also provided ad hoc reporting for use at the laboratory level. These reports are printed to provide a snapshot of TAT reporting and are suited for staff working at the laboratory level. At a national level, the corporate data warehouse (CDW) of the NHLS collated global TAT data from over 266 testing laboratories based on predetermined, annual performance plan cut-offs. National TAT cut-offs are set by expert committees of different pathology disciplines with final confirmation from senior management before implementation. These cut-offs are set with provisions for all levels of service laboratory: from low-volume laboratories with limited test repertoires to high-volume testing laboratories with extensive test offerings, including specialised testing, such as viral load testing. However, a large percentage of NHLS laboratories have 24-hour service and have emergency units in the hospitals in which they are housed; such laboratories have locally stricter TAT cut-offs for emergency and other local tests than are reflected in the national cut-offs for all samples.

Historically, the NHLS CDW TAT reports generated were static and reported only the mean TAT. Turn-around time data have a positive skewness, that is, a long tail to the right, meaning that the mean will be greater than the median. This implies that TAT data reported previously,^1,3^ reporting the mean TAT, masks good performance, while concealing poor efficiency. Further, neither the current LIS nor CDW reports enable detailed analysis of the information or drilling down to laboratory or test level data for additional information about TAT efficiencies. Data presented at the first conference of the African Society for Laboratory Medicine in Cape Town, South Africa, in 2012, reported daily laboratory test volumes and mean TAT for authorised results, stratified by individual laboratories,^4^ providing a snapshot of performance. This enabled review of CD4 laboratory efficiency for a national programme and provided important insights into laboratory operations.

A recent evaluation of TAT for HIV programmes reported a methodology to further categorise laboratory TAT performance using three additional measures:^3^ (1) median TAT, (2) 75th percentile TAT (tail size) and (3) percentage within cut-off TAT. These data were graphically presented using a scatter plot of percentage of samples within the TAT cut-off (*x*-axis) against the TAT 75th percentile (*y*-axis), categorised into four quadrants of performance to help identify the level of laboratory performance in a national programme. This approach made it easier to identify both good performers and outliers in the same analysis.^3^ The report was generated in Excel and included the raw monthly data, a scatter and bar graph and a summary table of all laboratories per business region, the 75th percentile, the percentage within cut-off values and TAT component 75th percentile values. This report was primarily distributed to managers of all HIV-related operational programmes, that is, CD4, viral load and tuberculosis testing and early infant diagnosis for review and intervention, and not shared across the network of testing laboratories.

Senior management in Gauteng, South Africa, expressed a need for a TAT monitoring tool that would enable them to better manage their laboratories and identify sites with poor TAT performance. Given the static nature of the historic TAT reporting in the organisation, an interactive system that offered information to enable review of performance, including outliers (tail size assessment), while confirming that sites were meeting cut-offs, would be a useful tool to enable business and laboratory managers alike to monitor their efficiency via TAT performance in real time. The concepts already developed and in use as Excel reports for the HIV programmes were the starting point for developing a reporting dashboard for use across multiple disciplines and tests done throughout the network of 241 testing laboratories of the NHLS in South Africa.

The aim of this study was to develop an easy-to-use information system, in a dashboard format. This would enable weekly reporting of TAT data as a snapshot of performance. To achieve this, a number of changes to current TAT reporting had to be addressed. These changes included (1) moving from programme-specific, single-test TAT reporting previously used^3^ to a specific set of high-volume tests, (2) adopting TAT measures reported by Coetzee et al.,^3^ (3) identifying dashboard software to use and (4) identifying the target users.^3^ The specific set of tests (or ‘basket’ of commonly requested tests) should be representative across the primary pathology disciplines.

This article sets out to describe the process followed to develop the TAT dashboard, using available software, that could provide a weekly summary of national, business unit and laboratory level TAT performance for a basket of tests. For the purposes of this article, data from a single participating tertiary laboratory were used to illustrate the data distribution, as it represents an example of a testing facility that performed all tests reviewed in the prescribed national basket. This was done to show the respective levels of drilling functionality of the dashboard and to iterate the interactive properties, while demonstrating how the dashboard can be used to assess performance and identify outliers for intervention.

## Methods

### Ethical considerations

Ethics clearance for this study was obtained from the University of the Witwatersrand (M1706108). Only anonymised laboratory data were used for the study and did not contain any patient identifiers.

### Study design

A retrospective descriptive study design was used to analyse and report laboratory TAT data for a specific set of tests (Table 1).

**TABLE 1:** Sample of a turn-around time dashboard table that lists the outcomes for the basket of tests, South Africa, 2018.

Test method name	Total tests	TAT target	% within TAT cut-off	75th percentile TAT (hours)
Activated partial thromboplastin time	333	5	100.0†	1
Alanine transaminase (ALT)	1655	8	99.0†	3
C-Reactive protein	1724	5	96.5†	3
CD4 ARV	3809	40	89.2†	26
Creatinine (plus MDRD)	8857	5	97.8†	2
D-Dimer	129	24	90.7†	14
Full blood count	4341	8	100.0†	1
Genexpert ultra	508	40	99.0†	6
Glucose (fasting)	25	5	95.0†	4
Glucose (random)	120	5	92.5†	3
HIV viral load	19 055	96	99.1†	55
HIV-1 qualitative PCR	495	96	99.2†	63
INT normalised ratio (INR)	952	3	99.1†	1
Platelet count	156	8	100.0†	1
RPR (syphilis)	52	36	83.8‡	35
T. pallidum antibodies	1013	12	92.2†	8
Total bilirubin	1135	8	97.9†	3
Total cholesterol	1240	5	86.0†	3

Note: The test method name, test volume, national stipulated cut-off turn-around time, percentage within turn-around time cut-off and 75th percentile turnaround time are reported. In this example, one test exceeds the national cut-off and local turn-around time (TAT) cut-off. This test requires corrective action to meet local TAT requirements and improve service delivery (e.g. rapid plasma reagin – syphilis). All other tests TAT outcomes are within local stipulated TAT for a tertiary 1000-bed hospital with an emergency unit, an Intensive Care unit (ICU) and specialist medical units. Local TAT cut-offs may vary from national TAT and are set based on local clinical requirements (local TAT targets are therefore better represented in the 75th percentile outcomes reported).

TAT, turnaround time; CD4 ARV, cluster of differentiation 4 antiretroviral; PCR, polymerase chain reaction; INT (INR), International normalised ratio; RPR, rapid plasma reagin; MDRD, Modification of Diet in Renal Disease Study equation.

† , ≥ 90% within TAT cut-off.

‡ , < 90% within TAT cut-off.

### Steps to developing a turn-around time dashboard

**FIGURE 1:**
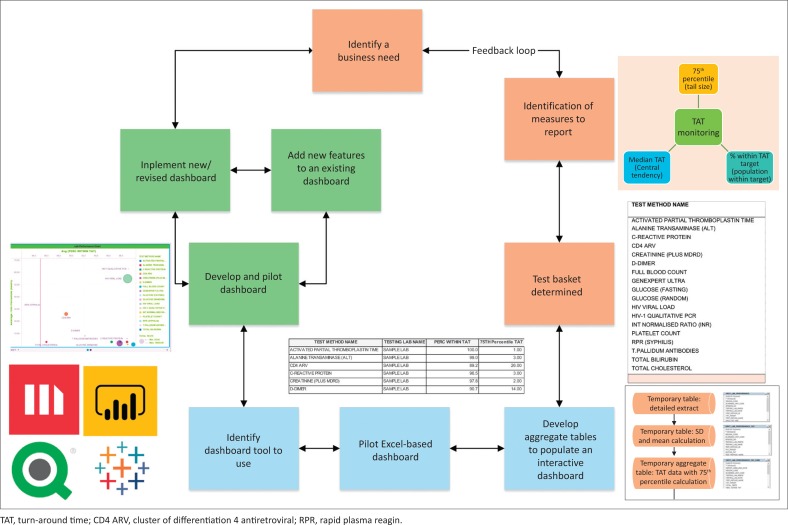
Flowchart depicting all steps required to develop a turn-around time dashboard, South Africa, 2018.

The various steps required to develop the dashboard are summarised in a flowchart (Figure 1).

### Sample population and turn-around time definition

Using convenience sampling, data were selected from among the tests performed at a single busy academic laboratory in Gauteng for one week during 2018. Aside from global TAT reporting, the dashboard should adopt the three TAT measures reported by Coetzee et al.^3^ These include pre-analytical, analytical and post-analytical components of TAT components, namely: (1) the time from first registration at the source laboratory to registration of the referral at the testing laboratory (LAB-to-LAB TAT), (2) time from registration at the testing laboratory to results being populated by the LIS interface (TESTING TAT) and (3) time from result population by the LIS interface to manual review and authorisation by senior laboratory staff (REVIEW TAT).

### Test basket development and inclusion and exclusion criteria

Focus group meetings were arranged with local area and business managers to define a test basket for the dashboard. The principles adopted were as follows: (1) measure a limited number of tests with a focus on the tests with the highest volumes of tests performed, (2) measure data for the indicator analyte (as a proxy) for specific panel tests (for example, the creatinine test was used as an indicator for assessment of urea and electrolyte test performance), (3) use the annual performance plan TAT cut-offs and (4) deliver dashboard files via email (due to bandwidth constraints). All samples within this organisation test basket were included in the example analysis and included the most commonly requested tests selected from haematology, coagulation, HIV-tuberculosis and chemistry (Table 1). A mapping table was developed to identify the LIS test sets and items to be reported. For each test, the TAT cut-off was also stipulated. The mapping table was used to guide the data extract.

### Data extraction

**FIGURE 2:**
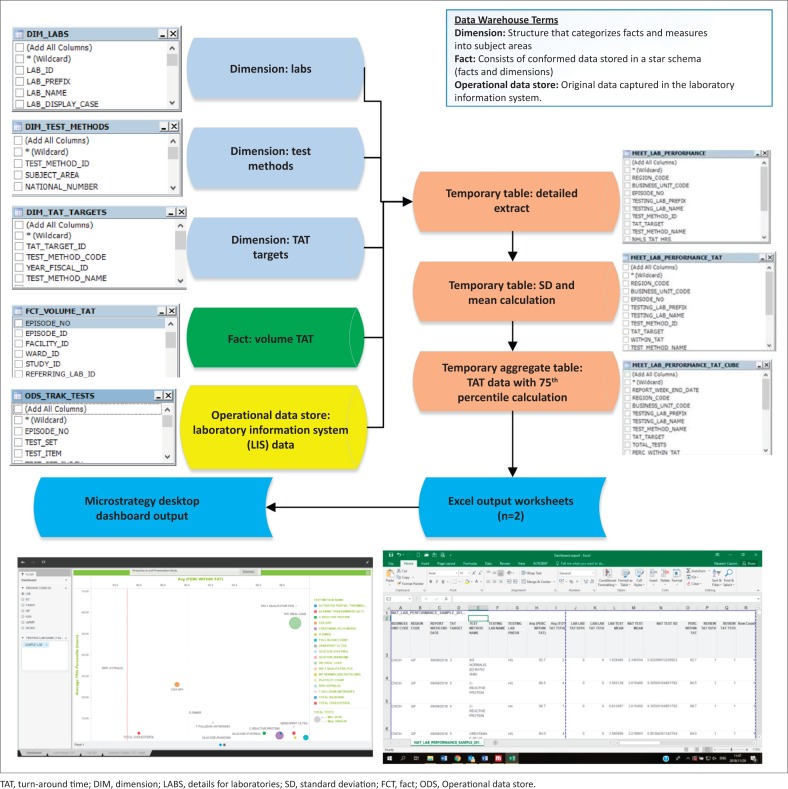
Visual representation of data preparation steps to transform and move raw turn-around time data, South Africa, 2018. Data were moved from the Operational Data Store (the laboratory information system) to a production server which consists of facts and dimensions. The production server structures the data by setting sets of data to specific target areas to facilitate final reporting in dashboard format.

For the purposes of demonstrating how the data were manipulated to create the dashboard, data were extracted for the week of 2–8 September 2018 from the CDW from four data sources: (1) the Operational Data Store that contained the original LIS data (Figure 2), (2) the ‘CDW fact’ that reported test volumes, (3) the test method dimension^5^ (provides details on the test such as a unique identifier, discipline, test method code and name and national number from the CDW) and (4) TAT cut-off dimension (captures annual performance plan cut-offs) (Figure 1).^6^ Using an outer join, data from these four data sources were prepared as a temporary detailed table. The first temporary table limited data to the test basket, adding the TAT cut-offs and provided information using the laboratory hierarchy (region, business unit and laboratory). Because this table would be too large to use for the dashboard and assuming email delivery of the final report, two additional steps were used to create a smaller aggregate data set. The mean, standard deviation, 75th percentile and percentage within TAT cut-off were added. All TAT data were reported in hours. The final temporary table was exported as a Microsoft Excel (Redmond, Washington, United States) worksheet and imported into the MicroStrategy Desktop analytics tool (Providence, Virginia, United States).^7,8^ After the data were imported, the respective dashboard sheets were developed to include relevant TAT information for all levels of management.

### Criteria for an effective dashboard

For any dashboard to be effective, it needs to adhere to a number of key outputs: it should (1) be visually engaging and easy to view and understand the TAT data displayed, (2) enable dynamic drilling down from a bird’s-eye view to a local perspective, that is, from national or provincial level down to the laboratory level per test, (3) provide a report on a weekly basis for a TAT snapshot view and (4) highlight TAT outliers for laboratory managers to follow-up and direct corrective action. From a more technical perspective, the dashboard also had to include additional features that included (1) conditional formatting to highlight good, average and poor performance, (2) provision of various reporting formats such as bubble charts, tables and bar charts, (3) ability to import data for a variety of formats and (4) ability to send the weekly dashboard data file via email, that is, small file format (≤ 6 MB).

### Data analysis and visual dashboard display

The dashboard displays (sheets) developed were as follows: (1) a bubble chart reporting the percentage within TAT cut-offs and 75th percentiles, (2) a table (Table 1) displaying the bubble chart data and (3) the 75th percentile for each phase of the component TAT reported by the test method. A bubble chart dashboard sheet was created to include: (1) the 75th percentile TAT (*y*-axis), (2) the percentage within TAT cut-off (*x*-axis), (3) the test volumes (size by and colour by) and (4) the test method (colour by and break by). The region codes and laboratory names were added to the dashboard as filters (radio buttons and search box display styles). An 85% within TAT cut-off reference line was added to aid identification of specific tests and associated laboratories with TAT that were outside of the TAT cut-offs. The data used to generate the bubble chart were also reported as a table in a separate sheet. The table listed the test name, total number of tests, TAT cut-off, the percentage within TAT cut-off and 75th percentile TAT for the basket of tests reported on.

The table uses ‘stop highlighting’ to denote the different percentage within TAT cut-off as follows: (1) 85% or higher in green, (2) 75% – 84% in orange and (3) under 75% in red. Lower percentage within TAT cut-off and higher 75th percentiles indicate an increased risk that any given laboratory is not adequately delivering patient reports that will enable timely clinical intervention.

A component TAT sheet was created as a clustered horizontal bar chart to display the component TAT as follows: (1) test method name (*y*-axis) and (2) component 75th percentile TAT (LAB-LAB TAT, TESTING TAT and REVIEW TAT), differentiated by colour. The testing laboratory name was added to enable refining and filtering data down to the laboratory level.

## Results

The successfully developed dashboard enabled delivery of weekly TAT data. Data from 45 599 reported samples for the week 2–8 September 2018 were utilised to demonstrate the dashboard development described here. The 75th percentile and the percentage of tests within stipulated cut-off for each test in the basket were visualised on the dashboard landing screen. This allowed the user to view data by test at the national, provincial and laboratory levels to visually identify outlying tests. The dashboard contained three individual sheets: the bubble chart, the TAT table and the component TAT sheets.

**FIGURE 3:**
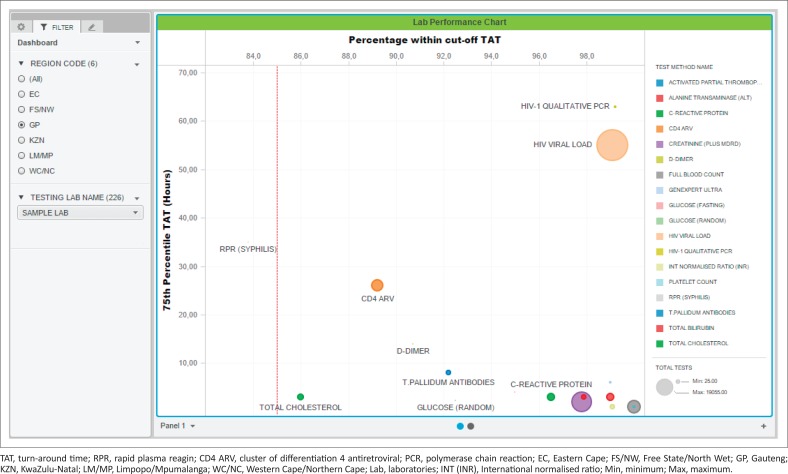
An example of the Microstrategy Desktop bubble dashboard chart used to report total turn-around time data for an example site’s week’s data, South Africa, 2018. The percentage within cut-off turn-around time is reported on the *x*-axis with the 75th percentile turn-around time on the *y*-axis. The bubble size indicates test volumes. Reference lines were added at 85% within stipulated turn-around time cut-off on the *x*-axis. Each test within the test basket is colour coded with the key provided on the right. Outlying tests are immediately visible.

Figure 3 shows typical weekly TAT data presentation outcomes as a bubble chart dashboard. In this example data set, only one test method, rapid plasma reagin (syphilis), failed to meet the 85% TAT cut-off and is reported as a small grey dot on the bubble chart dashboard. For this test, the reported percentage within cut-off was 83.8%, within the 75% and 84% category highlighted as orange in Table 1. A cluster of tests in the bottom right reported 90% or higher within TAT cut-off with a 75th percentile TAT of 8 hours or less. Only one test reported a percentage within TAT cut-off between 85% and 89%: total cholesterol (red dot). Higher test volumes were reported for the HIV viral load (*n* = 19 055) and creatinine (*n* = 8857) tests.

For the TAT table, results for the bubble chart are summarised (Table 1) per test. At the 75th percentile TAT, no test exceeded the cut-off TAT. A 100% within cut-off TAT was reported for three tests: activated partial thromboplastin time, full blood count and platelet count. Similarly, six tests reported a percentage within cut-off TAT between 95% and 99%.

**FIGURE 4:**
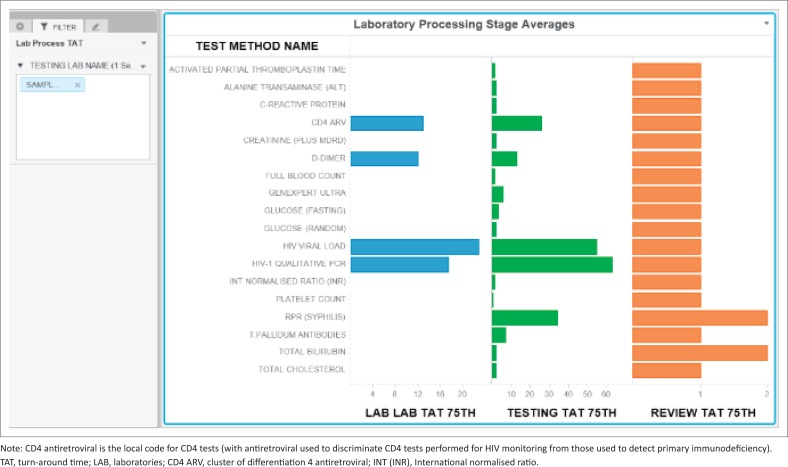
MicroStrategy Desktop dashboard bar chart used to report the component turn-around time data for an example site’s for the week 2–8 September 2018, South Africa. The components reported are LAB-LAB (inter-laboratory referral time), TESTING (time from registration to testing) and REVIEW (time from testing to review) turn-around time components.

The dashboard also reports component TAT in hours (Figure 4), including (1) LAB-TO-LAB, (2) TESTING and (3) REVIEW times, with the tail size in hours for the distribution of each component TAT. In any given laboratory, some samples tested are local (from the immediately adjacent hospital), while other samples are referred for testing from nearby hospitals where these tests are not available. As such, a zero LAB-TO-LAB component indicates that the samples were not referred but are samples collected and tested locally. For referred samples included in the example data set (see Figure 4, CD4 antiretrovirals, D-Dimer, HIV viral load among others), the LAB-TO-LAB component TAT 75th percentile represents the inter-laboratory referral time, ranging in this instance from 12 to 23 hours (Figure 4). In the testing phase, TAT ranged from 0.25 to 63 hours (where 63 hours represented a single test, the rapid plasma reagin, syphilis, that was regarded locally as an outlier; see Table 1 for detail). The 75th percentile review TAT was 2 hours or less across all tests.

## Discussion

Access to information in an interactive dashboard format has previously enabled retrieval of health data for immediate clinical use in the NHLS in South Africa.^8^ A similar approach has been applied and demonstrated in this work for TAT data. The dashboard described here provides an interactive, weekly snapshot of TAT performance together with information about TAT distribution, tail size (outlier) assessment,^1,3^ to varying levels of laboratory managers across the NHLS, to enable timely intervention where poor service delivery is identified.

The dashboard is comprised of a few basic parameters that act together to provide information about TAT. Date-stamping of samples in the LIS is a prerequisite to provide the basic information necessary to detail TAT linked to any given sample. Together with relevant sample identification datalogged, data is transferred to a central database for careful curation. Later TAT data extraction is performed using standard data query tools. In the instance of a wider network of laboratories operating within the same organisation, such as the South African NHLS, LIS data is stored using a decentralised architecture. Aggregate data, in the format described above, can then be collated and used to develop national TAT dashboards.

The dashboard described in this study simplifies presentation of complex data by enabling visualisation of any given laboratory’s efficiency. For the purpose of this study and to demonstrate the effectiveness and simple format of the dashboard developed, data from a single busy laboratory were used to illustrate the different outputs of the dashboard (graphs and table). The example data used here reveal how the dashboard can be used to identify tests that are not meeting national (or local) cut-off criteria. In the example presented, rapid plasma reagin (syphilis) testing was noted as an outlier as it did not meet the organisation-stipulated 85% within cut-off TAT. The summary table (example shown in Table 1) also provides a spreadsheet format table of the relevant tests either meeting, or failing to meet, the national cut-off criteria. The additional information on TAT component analysis further assists management to identify those areas of laboratory testing, within the respective pre-analytical, analytical and post-analytical components, that may need investigation for improvement.

The dashboard was successfully rolled out to all NHLS testing laboratories; weekly data are currently received by these laboratories for review. The dashboard development included a drilling down function into the performance of a particular test to see results by testing laboratory, or business unit. The addition of tail size measures^1,3^ has also enabled managers to identify less efficient areas of their laboratory services with outlying performance, seen in the example case described in this article (rapid plasma reagin – syphilis), which would have been otherwise missed using conventional reporting alone (using mean TAT reporting). In addition, to enable practical sample-by-sample audit, individual samples that did not meet cut-off criteria were identified for follow-up in an additional summary table sheet (added at the request of laboratory managers to enable better intervention, not shown). Use of the dashboard has also led to laboratory process changes with improved individual component TAT. For example, post-analytical TAT improvements included implementation of ‘auto review’.^9,10,11^ With respect to analytical delays identified, testing delays could be correlated with instrument breakdown logs from the laboratories or instrument suppliers to identify reasons for prolonged testing TAT.^9^ The impact of dashboard usage on improving TAT is described in detail in the companion article in this issue.^9^

Risk management teaches that not all errors can be predicted.^12^ However, it is only through active review of quality processes that delays, errors and problems can be detected earlier to enable corrective action. Thus, critical to managing risk is the continuous and ongoing evaluation and assessment of procedures and processes to ensure that the same errors are not repeated. Here, human capital is key to the sustainability and success of any dashboard implementation. Noble et al.^12^ reported that only the persistence and interest of laboratory personnel to maintain quality can ensure smooth and rapid progress of error detection (and correction back to quality) that is fast and sustainable. One of the fundamental lessons learnt from the development of the dashboard described here is that providing tools to assess TAT performance does not in itself imply corrective action or improvement. The dashboard is merely a tool that enables managers to effectively and efficiently ensure procedural excellence. Nkengasong et al.^13^ also suggest that in order for innovation to be adopted and sustainable, innovation and performance enablers should both energise and incentivise laboratories across four pillars: implementation, measurement, reward and improvement. A culture of diligence and willingness on the part of managers to meaningfully use information provided in the dashboard is thus important to enable making consequential changes at the laboratory level. Political will and strong senior leadership are also needed to make systems, such as those introduced with the dashboard described here, both functional and sustainable.^13^ This can be done by appropriately recognising and rewarding laboratories and personnel who use the tools provided.

Pre-analytical errors should not be underestimated, as they can increase both testing errors and TAT.^13^ In the example laboratory performance reported here, all four referred tests TAT outcomes were compromised due to pre-analytical delays. Documenting these delays and acting to reduce pre-analytical time, including travel time, and time spent in receiving centres prior to sample registration, can be used to streamline services.

Another outcome reported by managers using the dashboard was that the information could be documented week-by-week to provide objective evidence to document and motivate for additional resources required to achieve TAT cut-offs, for example additional sample collection schedules, increasing testing capacity, and motivation for auto review and authorisation modules.^9^

It is important in the context of a resource-poor setting to highlight that the dashboard described here was developed without specific funding, relying only on the collaborative effort of NHLS staff (the authors) with data management or MicroStrategy skills. Data is routinely transferred from the LIS to the CDW, where is it collated and carefully curated for downstream research and operational needs. Initial formats were undertaken using CDW extracted data analysed in MS Excel to create simple charts plotting 75th percentile and median TAT, by laboratory, for annualised or quarterly aggregated TAT data. Thereafter, analyses were extended to create week-by-week practical and usable worksheets so that individual laboratories could view current data. Using MicroStrategy, a freely available software program, a dashboard was developed to enable automatic presentation of the data in a visible interactive format (with the snapshot aggregate data file emailed to users weekly) to facilitate automated more immediate access to current TAT data. Future planning includes providing live data in the dashboard, facilitated by extending local bandwidth capacity and immediate real-time analysis of data within the CDW itself.

### Conclusion

This article outlines the database management and methods used for the development of a dashboard that enables presentation of weekly TAT data to relevant business and laboratory managers, as part of the overall quality management portfolio of the organisation. This novel approach ensures the delivery of quality, timely pathology reporting by the South African NHLS and, ultimately, better patient care. Training on the use of the dashboard is essential to ensure that users are competent. Users need to both understand the principles applied in the dashboard as well as the functionality embedded in the dashboard. Political will and leadership are vital to ensure that deficiencies identified by the dashboard lead to better quality and more efficient and timely laboratory services.

As African laboratories move toward increasing the number of centres that prepare for or achieve accreditation,^13^ it is vital that laboratories are aware of the commitment needed to continually monitor, evaluate and re-assess their status quo. Such commitment will ensure that the quality of the laboratory services they offer shows improvement over time. It is therefore important to consider what is required to achieve and maintain the quality of testing to avoid costly pitfalls^14^ and inaccurate or delayed result reporting. In this regard, although much of the focus of quality management is placed on quality of tests themselves, time management in a laboratory is as crucial as assuring the quality of the tests performed. Without timely delivery of patient results, appropriate and meaningful clinical management of patients cannot be accomplished.

### Limitations

The data presented in this study focus on the within-laboratory network TAT and did not record or assess delays outside the laboratory capture net. Pre-analytical TAT referred to in this work denotes the time taken to transport a sample from a receiving laboratory to a testing laboratory. Ideally, sample tracking systems that relay tracking data to the central data warehouse, linked to discrete samples,will enable total end-to-end service assessment of TAT.

Lessons from the field

- A data warehouse environment is able to collate national laboratory information system data to create a single weekly turn-around time report across a national laboratory service. This environment makes it easy to generate the aggregate data required.
- Dashboard development is an iterative process where feedback is essential.
- Dashboards can be developed in low- and middle-income countries using free or open-source data warehouse tools (provided that LIS data are generated).
- The use of dashboard tools that minimise bandwidth usage is optimal in an African context.
- Training is essential to ensure that users understand both the principles applied in the dashboard as well as the functionality embedded in the dashboard (e.g. drilling down and multiple worksheets).
- Ultimately, a true real-time dashboard, that could be used routinely by laboratories to identify and address outliers, would be ideal.
- A mandate from management to support the dashboard and facilitate correcting deficiencies identified is key to obtain support and buy-in at lower levels of management.

The dashboard subsequently developed has been extended to the top 22 highest volume tests performed across the organisation but does not report data for pathology sections like Microbiology or Anatomical Pathology disciplines or the more specialised units like Cytogenetics or Immunology. Plans are underway to broaden the test basket and to additionally include critical tests such as cardiac troponin levels, shown in other work (not reported here) to have TAT that currently falls beyond meaningful clinical impact.

The data presented provide only a weekly snapshot. As technology permits, it is important to extend and broaden development of this dashboard at the database warehouse level using business intelligence analytics tools that enable reporting real-time data. It is envisaged that laboratories could use large screens within laboratories themselves to track real-time progress for immediate response and corrective action, where required. Alternatively, remote management could be facilitated using specially developed mobile devices to display live TAT performance.

The currently reported dashboard data does not distinguish between different levels of service (i.e. tertiary versus primary and secondary hospitals) with different levels of patient care (intensive care unit, STAT-lab, trauma departments). Data is aggregated and compared to the national cut-off for each test in the dashboard presented here. However, individual laboratories have established locally-relevant TAT cut-offs for emergency and routine contexts depending on the level of care (primary versus tertiary).
